# Microbial and Geochemical Variability in Sediments and Biofilms from Italian Gypsum Caves

**DOI:** 10.1007/s00248-025-02576-3

**Published:** 2025-07-24

**Authors:** Tamara Martin-Pozas, Daniele Ghezzi, Ilenia M. D’Angeli, Giuliana Madonia, Veronica Chiarini, Marco Vattano, Jo De Waele, Martina Cappelletti, Cesareo Saiz-Jimenez, Valme Jurado

**Affiliations:** 1 Instituto de Recursos Naturales y Agrobiologia, IRNAS-CSIC, Seville, 41012 Spain; 2 Dipartimento di Farmacia e Biotecnologie (FaBit), Bologna, Bologna, 40126 Italy; 3 Istituto Italiano di Speleologia, Bologna, 40126 Italy; 4 Dipartimento di Scienze della Terra e del Mare, Università degli Studi di Palermo, Palermo, 90123 Italy; 5 National Biodiversity Future Center (NBFC), Palermo, 90123 Italy; 6 Dipartimento di Geoscienze, Università di Padova, Padova, 35131 Italy; 7 Le Taddarite Naturalistic and Speleological Association, Palermo, 90141 Italy; 8 Dipartimento di Scienze Biologiche, Geologiche ed Ambientali, Università di Bologna, Bologna, 40126 Italy

**Keywords:** Karst, Befana Cave, Re Tiberio Cave, Santa Ninfa Cave, Gypsum caves, Wall biofilms, Microbial aggregates, *Crossiella*, Wb1-P19, *Campylobacterota*

## Abstract

In Europe, several gypsum karst regions occur among which the gypsum karsts located in Emilia-Romagna (UNESCO World Heritage Site since 2023) and Sicily are notable. The sediments, spring water microbial aggregates, and wall biofilms from three caves, Re Tiberio, Befana (Emilia-Romagna), and the Sicilian Santa Ninfa, have been studied from a microbiological and geochemical point of view. The samples of wall biofilms from gypsum caves were exclusively composed of *Bacteria*, while the sediments showed negligible abundances of *Archaea*. The two most abundant phyla in most sediments and biofilms were *Actinomycetota* and *Pseudomonadota*, whereas the microbial aggregates floating in the spring waters of Befana Cave showed a deviation from the typical abundance pattern as *Campylobacterota* replaced *Actinomycetota*, and the abundances of *Bacteroidota* and *Desulfobacterota* were high. The most abundant genus in the wall biofilms was *Crossiella* (*Actinomycetota*), but it was absent in the water aggregates collected in Befana Cave. The abundances of *Crossiella* in the cave sediments were very low. The dominant genera in Befana microbial aggregates showed different abundances and microbial composition when compared with the previously studied Frasassi and Fetida thermal sulfuric acid caves, located in Central and South Italy, respectively, suggesting that the community composition of the microbial aggregates is specific for each cave and related to the geochemistry of the sulfidic spring. Also, a different microbial community composition was found in Befana water aggregates with respect to the wall biofilms from Befana and Santa Ninfa caves. In the case of sediments, they significantly clustered together indicating that the microbial communities associated with sediments are similar, independently from the cave and possible other environmental parameters.

**Supplementary Information:**

The online version contains supplementary material available at 10.1007/s00248-025-02576-3.

## Introduction

Karst landscapes are characterized by unique geological formations such as sinkholes, caves, and springs formed through the dissolution of the soluble bedrock by natural waters [1]. These soluble rocks, mainly carbonates and evaporites, occupy about 15% of the land surface ice-free land area [2]. Carbonate rocks, such as limestone and dolomite, form the most extensive karstic caves, but karstification also occurs in evaporite rocks such as gypsum creating diverse gypsum landscapes across different regions (Argentina, Cuba, Italy, New Mexico, northern Russia, Spain) [3]. In Europe, several notable gypsum karst regions with caves exist, including the gypsum karsts located in Sicily, Calabria, Emilia-Romagna, and Piedmont in Italy, as well as in southern Spain and western Ukraine [4–6].

The microclimatology, hydrogeology, and geomorphology of Italian gypsum caves are well documented [4, 7–13]. From a geochemical perspective, gypsum caves differ significantly from limestone karst systems. Gypsum caves are generally more dynamic and fragile ecosystems where dissolution processes occur faster than in limestone karst. In gypsum caves, the dissolution of bedrock supplies calcium ions (Ca^2^⁺) and sulfate (SO₄^2^⁻) to the cave drip water. In contrast, in limestone caves, the dissolution of bedrock predominantly releases (Ca^2^⁺) and bicarbonate (HCO₃⁻) into groundwater. Furthermore, gypsum caves are very different from carbonate karsts, not only from a geochemical point of view (the first having high concentrations of dissolved sulfate), but also because of the buffering effect of the latter (gypsum caves show higher ranges in pH, whereas carbonate karst are always close to neutral). The amount of dissolved sulfate and pH values are known to be environmental factors strongly influencing microbial community structures and composition in subterranean environments [14, 15].

The interaction between bacteria and sulfate has mostly been described in caves formed by sulfuric acid speleogenesis (SAS) (e.g., [14, 16–19]). However, these caves primarily occur in carbonate rocks and are hypogenic formed by hydrogen sulfide-rich water rising from depths within the Earth and typically characterized by high-temperature conditions, while gypsum caves are epigenic, resulting from undersaturated rainwater-induced dissolution of the underlying rock and generally showing lower temperatures [20]. However, limited research has focused on the geomicrobiology of these karsts.

To our best knowledge, only a few records on the microbiology of Italian gypsum caves are available in the literature. Cacchio et al. [21] analyzed the speleothems in a Calabrian gypsum cave for evidence of organic origins, identifying bacteria such as *Bacillus, Burkholderia*, and *Pasteurella* spp., among the isolates, which were able to precipitate calcite in vitro and may have played an important role in the deposition of cave minerals. Messina et al. [22] reported that Monte Conca Cave in Sicily is a gypsum karst system that includes an active sulfidic spring with floating white filamentous mats on the water and surface sediments. The authors suggested that sulfur-oxidizing bacteria may form the foundation of an autotrophic system with a complex associated food web. In further studies on the same cave, Davis et al. [23] suggested that the prevalence of primary sulfur oxidizers, such as *Sulfurovum*, *Sulfurimonas*, *Thiovirga*, and *Arcobacter*, increased the production of organic carbon chemosynthetically, and Nicolosi et al. [24] revealed the existence of a complex food web associated with a sulfidic pool and chemoautotrophic microbial activity, including arthropods, gastropods, and diplopods. In the Emilia Romagna region, D’Angeli et al. [25] analyzed the geochemistry and microbiology of cave waters in the gypsum karst aquifers over a 5-year period (sinking streams, rivers within caves, and resurgences). The authors aimed to identify potential pollution sources and concluded that fluctuations in fecal microorganisms correlated with seasonality, local precipitation events, and bat biological activity.

Although previous studies have provided valuable insights into the microbiology of gypsum caves [12, 22–25], significant gaps remain in understanding the microbial biodiversity across different gypsum cave environments. While some research has focused on sulfidic systems, the microbial communities inhabiting epigenic gypsum caves, particularly those in Italy, remain largely unexplored. Expanding knowledge of these microbial communities is crucial for understanding their biogeochemical significance.

The main aim of this work is to characterize the microbial communities involved in the sediments and white biofilms on walls and microbial aggregates from spring waters within these Italian gypsum caves. This study focuses on three caves from the most significant and well-studied Italian gypsum karst areas: Grotta del Re Tiberio and Grotta della Befana, located in the Messinian gypsum formations of Emilia Romagna (which became a UNESCO World Heritage Site in 2023), and Grotta di Santa Ninfa (protected as a Nature Reserve) in Messinian gypsum formations of Sicily [5, 9, 26]. Although these caves share a common geological epigenic origin, they exhibit distinct hydrologic characteristics. Befana Cave is characterized by the presence of a sulfidic spring inside the cave that rises from the lower lying evaporite layers. Sulfate reduction occurs in deeper layers of the aquifer where oxygen depleted conditions persist, also thanks to the presence of organic matter. In Santa Ninfa Cave, a sulfidic spring was reported [27], but currently is no longer present, while in Re Tiberio Cave no evidence of the presence, at least in the recent past, of this kind of spring was found in the sampled area. This study provides new insights into the microbial diversity of these gypsum karst regions, contributing to a broader understanding of microbial subsurface life.

## Material and Methods

### Sampling

A total of 16 samples were collected in 2022 from Re Tiberio, Befana, and Santa Ninfa caves (Table 1, Supplementary Figures S1–S4). Of these, nine samples were obtained from sediments, four from the white biofilms covering the walls and three from microbial aggregates floating in the spring waters. Sixteen more samples of cave sediments were also collected and analyzed geochemically (Supplementary Table S1).

**Table 1:** Caves, samples, and environmental parameters^1^

Cave	Sample	Type	T °C	RH %	CO_2_ ppm
Re Tiberio	TB-1A	Sediment	14.7	99.9	955
	TB-3C	Sediment	13.3	99.9	5632
	TB-8H	Sediment	13.3	99.9	2420
Befana	BF-3C	Water microbial aggregate	13.6	99.9	1190
	BF-6F	Sediment	13.7	99.9	940
	BF-7G	Wall Biofilm	ND	ND	ND
	BF-8H	Sediment	ND	ND	ND
	BF-05	Water microbial aggregate	13.3	ND	ND
	BF-19	Water microbial aggregate	13.0	ND	ND
Santa Ninfa	NF-1A	Sediment	16.5	94.5	1881
	NF-2B	Wall Biofilm	16.5	94.5	1881
	NF-3C	Wall Biofilm	16.1	99.9	2010
	NF-4D	Sediment	17.4	95.2	2068
	NF-5E	Sediment	17.0	99.9	2199
	NF-7G	Sediment	17.2	99.9	3471
	NF-9I	Wall Biofilm	16.1	99.9	2166

^1^For sample locations, see Supplementary Figs. 1, 2, and 3

Samples were collected using sterile scalpels. The preservation methods varied based on the type of analysis. Samples intended for DNA sequencing and molecular analysis were preserved in Lifeguard preservation solution (Qiagen, Hilden, Germany) until their arrival at the laboratory, where they were stored at − 80 °C. For geochemical analyses, sediment samples were collected in sterile tubes and preserved at 4 °C.

In Re Tiberio Cave (thereafter Tiberio), located 500 m South of Borgo Rivola, adjacent to the still active gypsum quarry of Monte Tondo, we sampled sediments along the main cave branch, in the historical branch and Gothic Hall (180 m asl, sample TB-8H) and in the main branch up to the “Baldo” crawlway (192 m asl, samples TB-3C in the middle (Supplementary Figure S1A) and TB-1A at the end of the so-called speleological branch, before the passage size becomes very narrow (“Baldo crawlway”) [28, 29] (Supplementary Figure S2). These underground river passages have formed during MIS6 (185–135 ka), when the main cave gallery was acting as a resurgence [13, 30]. At present, these cave environments are dry (i.e. no flowing water), but actively subdued to periods in which condensation occurs, when cave temperatures (around 11 °C) and those outside are very different (much colder or hotter). In Tiberio, the air temperature is around 13–14 °C and average CO_2_ levels range between 955–5,632 ppmv (Table 1). Several rooms along these branches were roosting places for large bat colonies in the past, as old guano deposits indicate, but today bats are rarely observed. The entrance portions of the cave have been used as burial place during the Copper and Early Bronze age (ca. 5 ka), and later as a shelter [31]. During WWII the initial parts of the cave (Gothic Hall) were also used as bomb shelter by groups of local inhabitants. Today, the first 80 m (where sample TB-8H was taken) is open to public (ca. 3000 visitors/year), whereas the main branch is occasionally visited by small groups of visitors [32]. No conspicuous biofilms were found on the walls of this cave.

Befana Cave (here after Befana) develops parallel to a North-facing gypsum ridge, upstream an abandoned gypsum quarry (ex Cava Paradisa) half a kilometer West of Borgo Tossignano (Supplementary Figure S3). In Befana**,** the air temperature is around 13–14 °C and average CO_2_ levels range between 940 and 1190 ppmv (Table 1). The cave has several entrances, some caused by the nearby quarry. One of the central entrances (opening at 210 m asl) is a narrow shaft that leads into a large room 30 m lower. This chamber reaches a small river 10 m lower, flowing from West to East. At around 160 m asl a sulfidic spring is encountered, characterized by the typical rotten egg smell and by white masses of microbial aggregates (streamers) in the water. This is where samples BF-3C and BF-05 were taken in the water (Supplementary Figure S1B). Another sulfidic spring is located 40 m downstream and was also sampled (sample BF-19). The other sediment samples were taken along the inclined cave passage, in the sediment banks besides and a few meters above the river and at the entrance in the room, 10 m above the cave river (BF-6F and BF-8H, respectively). Sample BF-7G concerns white spots and coatings on the mud-covered cave roof above the underground river, similar to biofilms described in other carbonate caves (Supplementary Figure S1C) [33].

The Santa Ninfa Cave (from here on Ninfa) is part of an active karst system roughly 1300 m long and about 30 m deep, located in Western Sicily (Supplementary Figure S4). The system developed at the end of a big blind valley and consists of a sink cave (“Inghiottitoio del Biviere”), accessible only for a few meters, Santa Ninfa Cave (“Grotta di Santa Ninfa”) and an impracticable resurgence. The average air temperature in the cave is roughly 16 °C and average CO_2_ level is around 2,000 ppmv [34]. The cavities are separated by sumps, but their hydraulic connection was confirmed by the use of tracers. A passage in a collapse zone allows access to the Santa Ninfa Cave. Cave passages are arranged in two main levels: in the upper inactive level the samples NF-1A, NF-2B, NF-3C, and NF-4D were collected. In the middle part of the upper passages, large ancient deposits of bat guano occur, linked to a bat colony not present anymore. The lower level is connected to the upper level by a short drop. The samples NF-5E and NF-7G were collected in the lower level where the passages are largely fed by an underground river coming from the sink cave and returning to daylight through the resurgence about 20 m below the present-day entrance. The NF-9I sample comes from the area where a sulfidic spring was reported in the past [27]. Samples NF-2B, NF-3C (Supplementary Figure S1D), and NF-9I concern white spots on the cave, similar to white spots described in lava caves [35]. For each sampling site CO_2_, T and relative humidity of cave atmosphere were recorded using a multiparametric device (measuring range: CO_2_ 0–9999 ppmv, res 1 ppmv; Temp. − 10–60 °C, res. 0.1 °C; humidity 0.1–99.9%, res. 0.1%) (Table 1).

### Geochemical Analyses

Sediment samples were prepared for analysis by first crushing and sieving them through a 2 mm mesh, followed by further grinding to achieve a particle size of less than 60 µm. The pH of the sediment samples was measured using a 1:2.5-sediment-to-water ratio extract. After shaking the mixture for 1 h, pH values were obtained with a glass electrode.

Geochemical parameters and trace element concentrations of sediments were obtained using different laboratory methods described by Martin-Pozas et al. [36]. For metal and trace element analysis, samples were digested with aqua regia in a microwave digestion system. Elemental quantification was conducted with a VARIAN ICP 720-ES. Analytical accuracy was ensured using the Community Bureau of Reference (BCR) standard reference materials, alongside reference soil samples from the Wageningen Evaluating Programs for Analytical Laboratories (WEPAL), and International Soil-analytical Exchange (ISE).

The water samples were collected at each site: 250 ml and 50 ml (sterile container) of normal water and 100 ml of water filtered with a 0.45 μm sterile filter (sterile cellulose acetate, Minisart©) and acidified with 1 ml of concentrated 65% HNO_3_. At each sampling site pH, temperature and electric conductivity were measured in situ with a previously calibrated Hanna HI991001 portable sensor (accuracy 0.02 °C, 0.5 pH, and 2% EC/TDS). Data were obtained in the same fieldwork session, concurrently with sample collection.

Geochemical analyses were performed at the Politecnico di Torino. The concentration of dissolved HS^−^ was measured in situ using a Hach DR/2010 spectrophotometer. Cations were analyzed using an atomic absorption spectrophotometer Thermo S (AAS), anions with ion chromatography, and alkalinity (HCO_3_^−^) through titration with HCl and methylorange as pH indicator.

### Microscopy

The microscopic study was performed in the Non-Destructive Techniques Service of the MNCN-CSIC, Madrid, Spain. Detailed pictures of white wall biofilms were taken using a digital microscope Dino-Lite (model Edge AM4115ZT). To prevent the collapse of the cells which form the white wall biofilms, a Field Emission Scanning Electron Microscope (APREO 2S) was used in Cryo mode at 2 kV. For this purpose, the samples were previously frozen in liquid nitrogen in a very short time to prevent ice crystal formation and preserve the native structure. Spring water microbial aggregates were visualized using Field Emission Scanning Electron Microscopy (FESEM), Environmental Scanning Electron Microscopy (ESEM) and confocal microscopy. Samples were previously fixed with 2% buffered paraformaldehyde and dehydrated with an ethanol series. An additional step of hexamethyldisilazane (HMDS) was added to preserve the structure for electron microscopy imaging. Samples were visualized in low vacuum conditions after coating with a thin conductive layer of gold particles to improve secondary electron emission, image contrast, and resolution. For confocal imaging, samples were stained with Syto Deep Red Nucleic Acid Stain to stain the DNA and visualized under a LEICA TCS SPE Spectral Laser Confocal Microscope. Negative controls were used to control autofluorescence.

### DNA Extraction, Sequencing, and Data Analyses

Genomic DNA extraction was performed using the FastDNA SPIN Kit for Soil (MP Biomedicals, Illkirch, France) for all the samples. DNA concentrations were quantified using a Qubit 2.0 fluorometer (Invitrogen, Carlsbad, CA, USA). High-throughput sequencing of the extracted DNA was conducted by FISABIO Institute (Valencia). The bacterial V3 and V4 regions of the 16S SSU rRNA gene were amplified using the primers Bakt 341 F and Bakt 805R, then were analyzed using Illumina MiSeq technology with 2 × 300 paired end sequencing. Sequence data were processed using the QIIME2 pipeline [37]. Metataxonomy analysis was performed using some of the QIME2 plugins. Denoising, joining of the paired ends reads, and chimera depletion were conducted using the DADA2 pipeline [38]. Taxonomic affiliations were assigned using the Bayesian classifier integrated within the QIIME2 plugins and pre-trained using the SILVA 138 SSU reference database for the V3–V4 region [39]. All computations and statistical analyses were performed within the R environment [40].

Taxonomic data obtained from QIIME2 were imported into R and analyzed using the phyloseq package. To assess ecological alpha diversity within each sample, we employed non-phylogenetic diversity Chao1, Shannon, and Simpson indices at species level calculated with the R packages phyloseq and vegan, with statistical significance determined by ANOVA. Beta-diversity was assessed at genus level using Principal Coordinates Analysis (PCoA) with Bray–Curtis distance and PERMANOVA statistical method. Plots and heatmap were generated using the R packages ggplot 2 and pheatmap.

## Results

### Geochemistry of Sediment Samples

The geochemical results highlight the diverse environmental conditions of sediments within the caves. Supplementary Table S1 shows that the sediments from the three caves have a pH ranging between 6 and 8, except Befana, which has acidic pH in two sediments. TB-3C is notable with the lowest pH (3.77), indicating a highly acidic environment. Organic carbon is low in most samples, but the sediment NF-7G reaches 1.02%, while TB-1A and the two samples of gypsum from Ninfa are very low. Carbonates (CaCO_3_) are low in Tiberio, and higher in Befana and most Ninfa samples, with the highest content in the NF-5E sediment (53.9%). On the contrary, sulfur is high in the two gypsum samples and NF-4D from Ninfa, and variable in the other sediments, with the lowest value in Tiberio TB-1A. Iron is low in the two gypsum samples and other sediments from Ninfa but comparatively higher in Befana and Tiberio. Nitrogen compounds and phosphorus vary considerably across the samples. TB-3C shows extremely high nitrate levels (1030 mg/kg) and phosphorus (309 mg/kg), distinguishing it from all other sediments. In contrast, ammonium concentrations are highest in NF-4D (52.5 mg/kg) and NF-5E (63.5 mg/kg). The CEC is low in the two Ninfa gypsum samples, as corresponds to gypsum low solubility, with the higher values in Tiberio, and low to medium in Ninfa sediments. Tiberio sediments exhibit the highest CEC, while Ninfa sediments show lower values. TB-6F and TB-3C present significantly higher CEC values (55.4 and 35.7 cmol/kg, respectively), indicating a greater capacity for nutrient retention compared to the other sediments.

### Geochemistry of Water Samples

The water samples in Befana are slightly alkaline showing values ranging between 7.04 and 7.33, and the temperature is close to 13 °C (Supplementary Table S2) [25]. Since the host rock is made of gypsum (CaSO_4_.2H_2_O), major elements are related to SO_4_^2−^ and Ca^2+^. Additionally, Na^+^ and Cl^−^ are abundant because halite is sometimes associated with gypsum due to the process of evaporation. The sulfides range between 7.3 and 36.2 ppm. The values of fluoride are above the guide value for human tolerance established at 1.5 ppm. The nitrate concentration in sample BF-19 (76.6 ppm) is also above the guide limit (50 ppm, according to the Drinking Water Directive 2020/2184). On the contrary, nitrates have not been detected in BF-05, highlighting the geochemical heterogeneity within the cave system. The total dissolved solids (TDS) values are high (3328.8–3907.1 ppm), indicating a high content of inorganic and organic substances dissolved in water.

### Microscopic Characterization of Wall White Biofilms and Water Microbial Aggregates

**Fig. 1:**
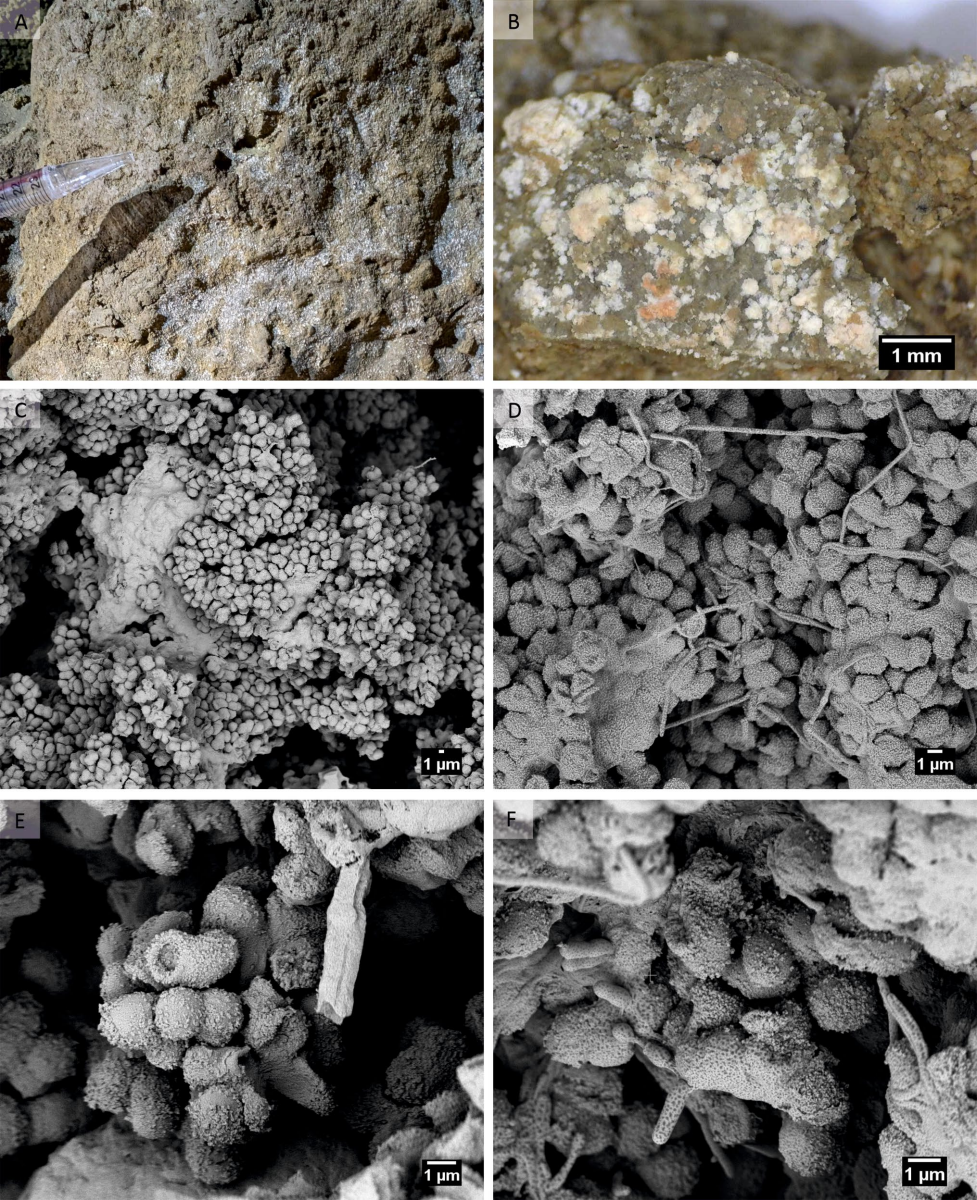
White wall biofilm. **A** General view of the biofilm in Befana Cave, and **B** under optical microscopy. **C**–**F** Cryo-field emission scanning electron microscopy (Cryo-FESEM) images of white wall biofilm. **C**, **D** Overview of the biofilm surface. **E** Detail of rounded cells. **F** Detail of rod-shaped bacteria and filaments

Figure 1 shows the most representative cellular structures of white wall biofilms.

Under optical microscopy, these biofilms showed a granular texture with irregular margins and variable sizes, from punctiform whitish spots to larger agglomerates. At higher magnifications, the most abundant structures were clustered rounded cells (1.5–1.7 µm) with ornamented surfaces, rod-shaped bacteria (0.4 × 1.8 µm), and filaments (0.4 µm diameter). No signs of mineralization were observed.

**Fig. 2:**
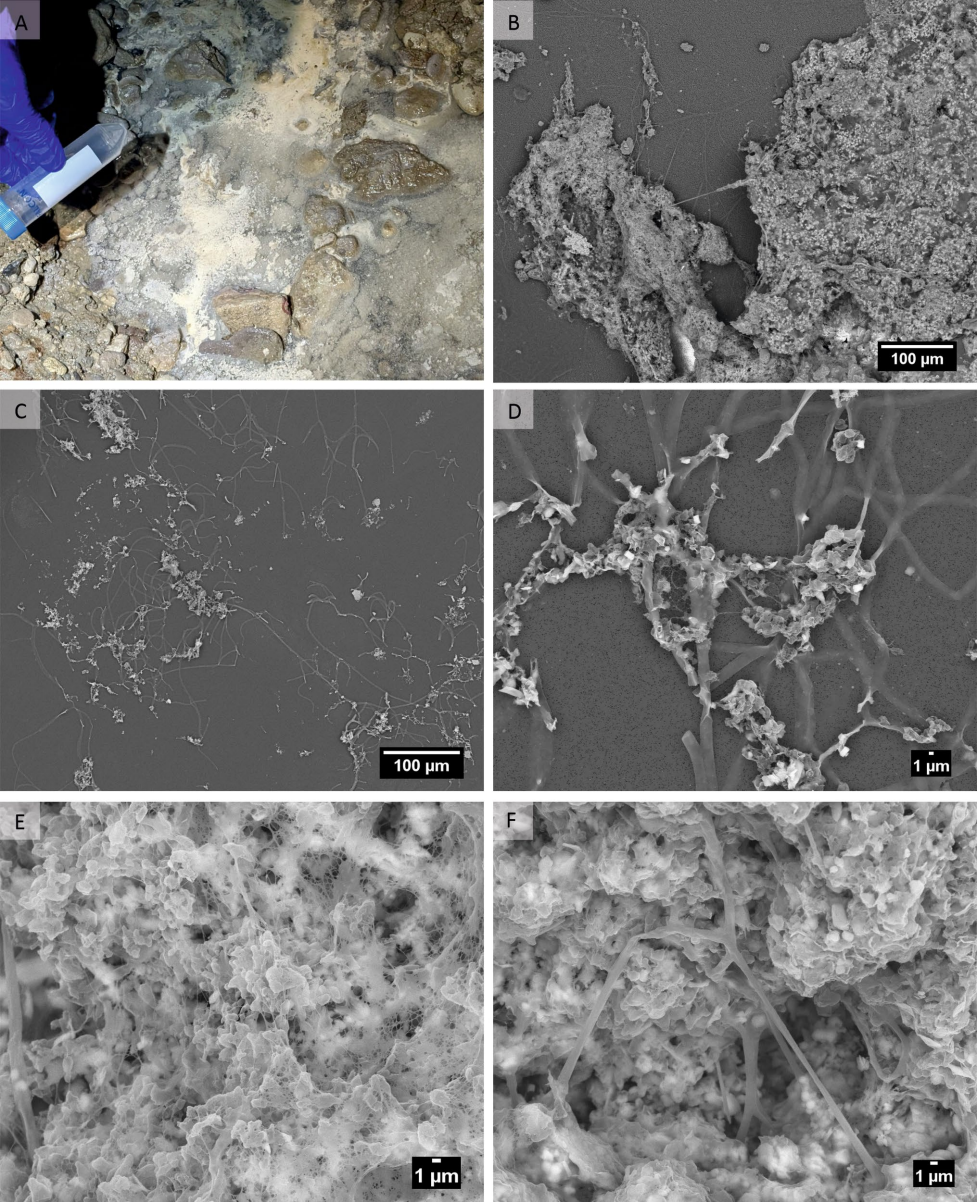
Water microbial aggregates. **A** General view of the aggregates in Befana Cave, and **B** under FESEM. **C**–**D** Detail of filaments. **E** Detail of microbial aggregates. **F** Detail of microbial aggregates and filaments embedded in the extracellular matrix

Field emission scanning electron microscopy (FESEM) showed that the water microbial aggregates were mainly composed of short rod-shaped or coccoid cells embedded in a dense extracellular matrix, sulfur, and lower abundance of filaments measuring 1–2 µm in diameter (Fig. 2). In contrast to wall biofilms, EDS analysis detected sulfur and calcium in the microbial aggregates (Supplementary FigureS5A-C). Confocal microscopy using Syto Deep Red staining, which labels nucleic acids, showed strong fluorescence signals corresponding to spherical structures (0.3–0.6 µm) located within the filaments and bacterial cells within the dense matrix (Supplementary FigureS6).

### Microbial Community Diversity

**Fig. 3:**
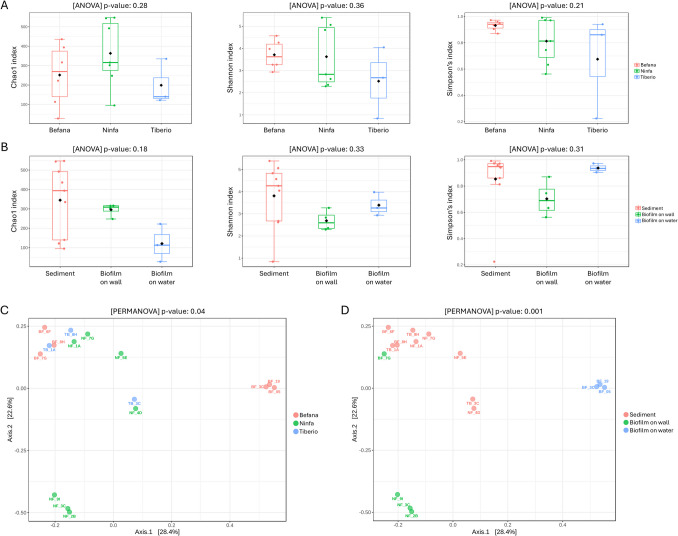
**A** Alpha diversity based on cave. **B** Alpha diversity based on type of sample. **C** Beta diversity based on cave. **D** Beta diversity based on type of sample

Alpha diversity analyses did not indicate significant difference between the samples grouped based on the cave or the type/nature of the sample (Fig. 3A–B). Chao1, Shannon, and Simpson indexes greatly varied between the different samples with two sediments NF-7G and NF-5E, both from Santa Ninfa, showing the greatest richness and diversity values (Supplementary Table S3). Beta diversity analysis (Fig. 3C–D) showed that the microbial samples clustered depending on the sample type, which is closely associated with the substrata where they grow: sediment, walls, water. The exception was the BF-7G sample, the white biofilm from the mud-covered Befana roof, which did not cluster with the white wall biofilms from Ninfa but clustered with the sediments from all the caves. This difference can be attributed to the fact that the BF-7G white biofilm grows on a mud substratum, whereas the other white biofilms grow on gypsum walls. In the case of sediments, which were collected from all the caves under analysis, they generally clustered together indicating that the microbial communities associated with sediments are similar independently on the cave environment (Fig. 3C–D). However, the sediments NF-5E and NF-4D from Ninfa and TB-3C from Tiberio appeared more distinct in the PCoA analysis, compared to the other sediments, which clustered together (Fig. 3D).

### Microbial Community Composition

The samples of white biofilms and microbial aggregates from the three Italian gypsum caves are exclusively composed of *Bacteria*, regardless of the substrata they grow on, while the sediments exhibited negligible relative abundances of *Archaea* (0–0.62%) (Supplementary Table S4).

**Fig. 4:**
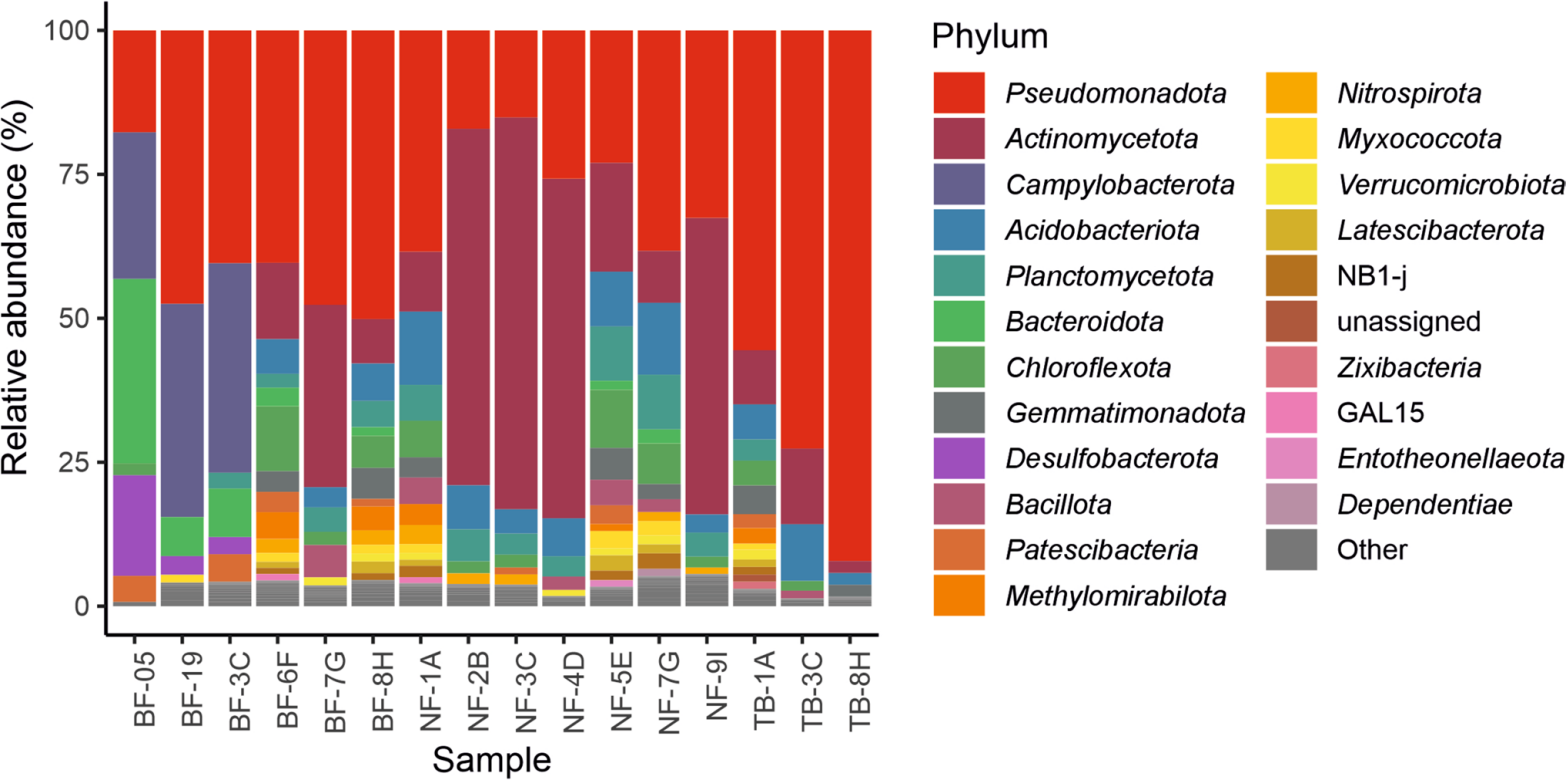
Bacterial relative abundance at the phylum level in gypsum caves. Phyla with relative abundances below 1% are represented as “Other”

Figure 4 compares the relative abundance of bacterial phyla in white biofilms, microbial aggregates, and sediments collected from the different caves. *Actinomycetota* and *Pseudomonadota* were the two most abundant phyla in most samples. *Actinomycetota* was generally more abundant in wall biofilms (31.67–68.06%), while *Pseudomonadota* was more prevalent in sediments (23.00–92.16%), particularly in Tiberio (55.54–92.16%). The exception is the sediment NF-4D, which presents an anomalously higher percentage of *Actinomycetota* compared to the other sediments.

However, three samples showed a deviation from the typical abundance pattern of *Actinomycetota* and *Pseudomonadota*. These samples were collected from the microbial aggregates floating in the spring water of Befana and showed high relative abundances (36.38% in BF-3C, 25.44% in BF-05, and 36.88% in BF-19) for *Campylobacterota*, replacing the *Actinomycetota* present in the other samples. In these aggregates the relative abundance of *Actinomycetota* was 0.59% in BF-3C and absent in the other two samples. Notably, *Bacteroidota* also exhibited relative abundances from 6.76 to 32.08% in the microbial aggregates, while *Desulfobacterota* were high in BF-05 (17.51%), and showed lower percentages in BF-3C and BF-19 (2.97% and 3.24%, respectively).

Other twenty-one phyla presented relative abundances greater than 1%, with the following phyla exceeding 5% in at least one sample: *Desulfobacterota*, *Acidobacteriota*, *Chloroflexota*, *Bacillota*, *Gemmatimonadota*, and *Plactomycetota*. *Desulfobacterota* dominate the spring microbial aggregates in BF-05 (17.51%), *Acidobacteriota* were generally more abundant in the sediments of the three caves (2.08–12.73%) compared to the white biofilms and aggregates (0.31–7.64%), *Chloroflexota* showed relative abundances of 0.11–2.28% in white biofilms and aggregates, and 0.38–11.29% in sediments. *Plactomycetota* was relatively important in Ninfa with relative abundances ranging from 3.52 to 9.50%. *Gemmatimonadota* presented abundances greater than 5% in two sediments from Ninfa and one from Tiberio. *Bacillota* was significant only in the white biofilm from Befana walls (5.63%), and was negligible in the water microbial aggregates (0.11%), as well as in the wall white biofilms of Ninfa (0.18–0.83%). *Bacillota* was low (0.19–1.33%) in Tiberio sediments and also those from Befana (0.34–0.85%), while its abundance increased in the sediments of Ninfa (2.22–4.62%).

**Fig. 5:**
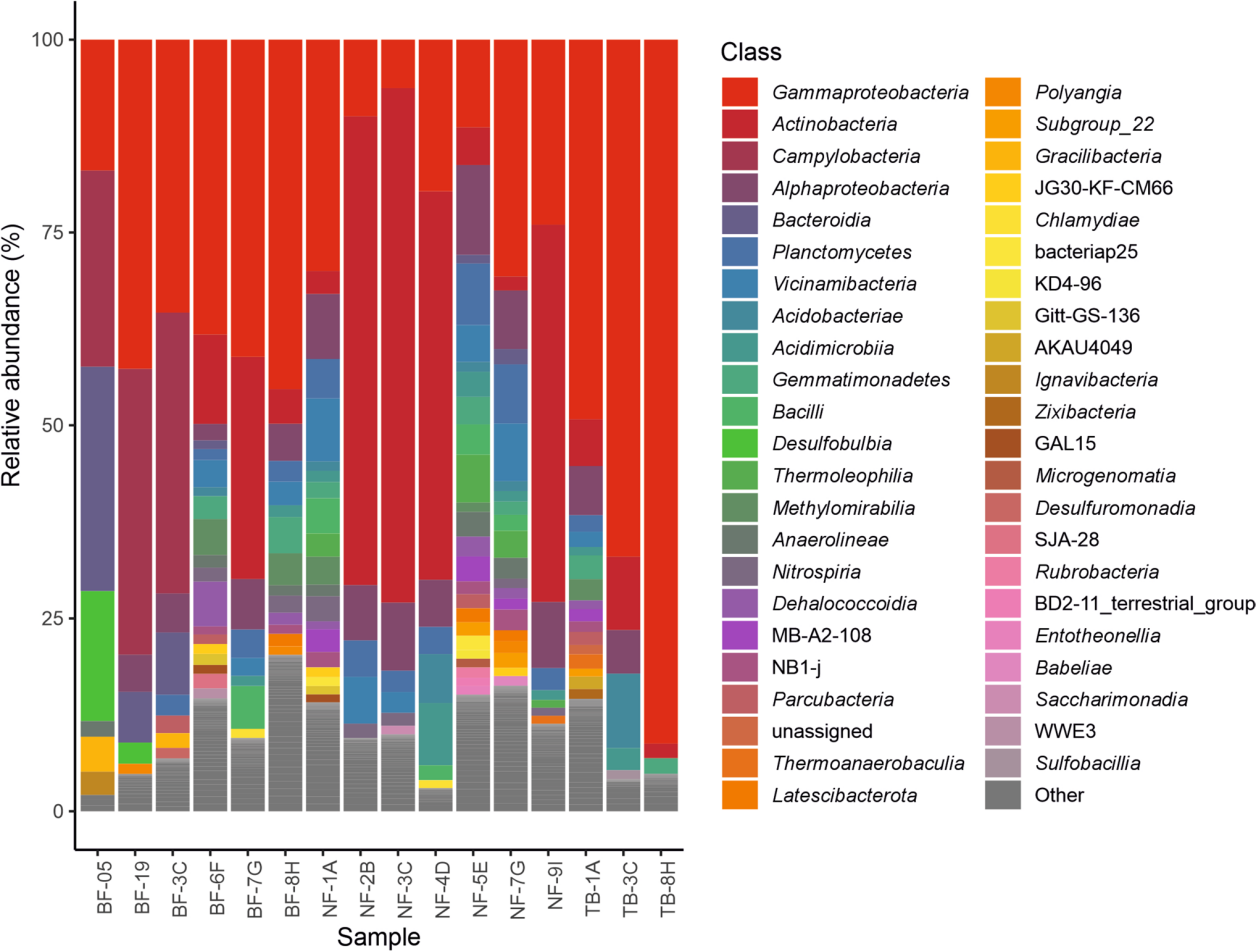
Bacterial relative abundance at the class level in gypsum caves. Classes with relative abundances below 1% are represented as “Other”

Only six bacterial classes showed > 10% of relative abundance: *Actinobacteria*, *Gammaproteobacteria*, *Alphaproteobacteria*, *Bacteroidia*,* Campylobacteria*, and *Desulfobulbia*, but the last two classes were retrieved from water microbial aggregates, and exceptionally appeared in Ninfa sediment NF-7G with negligible abundances of 0.02% and 0.01%, respectively (Fig. 5). The microbial aggregates showed high relative abundances of *Gammaproteobacteria* (16.96–42.51%), *Campylobacteria* (25.44–36.88%), *Bacteroidia* (4.85–29.06%), and *Desulfobulbia* (0.61–16.87%). The sediments showed variable relative abundances within caves and samples for *Gammaproteobacteria* (11.35–91.21%), *Actinobacteria* (1.82–50.35%), and *Alphaproteobacteria* (0.94–11.65%). The wall biofilms showed the abundant occurrence of *Actinobacteria* (28.78–66.67%), followed by *Gammaproteobacteria* (6.28–41.11%), and *Alphaproteobacteria* (6.55–8.56%). Seven classes (*Vicinamibacteria*, *Bacilli*, *Planctomycetes*, *Terriglobia*, *Thermoleophilia*, *Dehalococcoidia*, and *Acidomicrobiia*) showed relative abundances ranging from 5 to 10% with a high dispersion between caves and samples from the same cave.

**Fig. 6:**
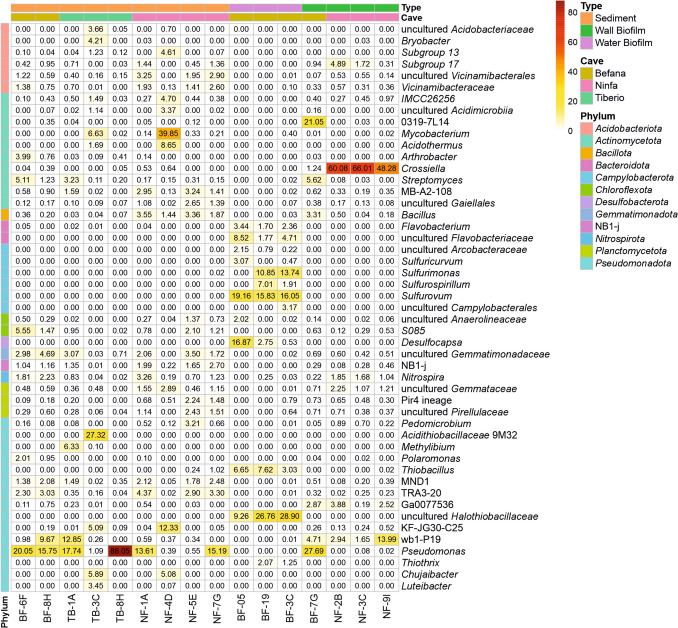
Bacterial relative abundance at the genus level in gypsum caves. The scale bar represents relative abundance, with white squares indicating the least abundant classified genera and red indicating the most abundant. Bars on the left and the legend on the right indicate classification at the phylum level. Genera with abundances below 2% were not included

From all the genera identified, 95 showed a relative abundance greater than 1% in at least one sample and only 19 genera presented relative abundances exceeding 5% in at least one sample. Figure 6 shows the genera identified with relative abundances greater than 2% in at least one sample, including those classified as uncultured. Some genera were more commonly found in sediments, while others were dominant in wall and water biofilms. Nevertheless, these genera showed a marked variability and dispersion across biofilms, sediments, and caves.

Within the *Actinomycetota* phylum, the most abundant genus was *Crossiella* with relative abundances ranging from 48.28 to 66.01% in the white biofilms from Ninfa, 1.24% in Befana, but absent in the water microbial aggregates collected in this cave. The abundances of *Crossiella* in the sediments of Tiberio were negligible (0.00–0.05%), very low in Befana (0.04 and 0.39%), and Ninfa (0.00–0.64%). Other abundant *Actinomycetota* genera in these caves included *Streptomyces*, *Mycobacterium*,* Acidothermus*, and lineage 0319-7L14. *Streptomyces* reached relative abundances greater than 5% in a sample of wall white biofilm and a sediment from Befana, with lower abundances (0.20–3.23%) in Tiberio and absent or low (0.00–0.31%) in Ninfa. *Mycobacterium* was very important in a sediment sample from Ninfa (39.85%) and Tiberio (6.63%), while it was absent or had low abundances (0.00–0.40%) in the other samples. *Acidothermus* was absent in all samples, except in a sediment from Ninfa (8.65%) and Tiberio (1.69%). 0319-7L14 was relatively important in the wall biofilm (21.05%) from Befana while it was absent or had low abundances (0.00–0.35%) in the other samples.

Among the *Pseudomonadota* stand out *Pseudomonas*, the *Nitrococcaceae* wb1-P19, the *Acidithiobacillaceae* 9M32, an uncultured *Halothiobacillaceae*, and the gammaproteobacterial KF-JG30-C25, which showed relative abundances greater than 10%. The *Comamonadaceae Methylibium*, *Rhodanobacteraceae Chujaibacter*, and an unidentified *Acetobacteraceae* showed relative abundances around 5%. *Pseudomonas* was abundant in most sediment samples (15.75–20.05%) and in the Befana wall biofilms (27.69%). In sediments from Tiberio, *Pseudomonas* reached the highest abundance 88.05%, decreasing in the Ninfa sediment samples, where the abundances were negligible in most of the sediments. It was absent in the water microbial aggregates and had low abundances (0.00–0.40%) in the Ninfa wall biofilms. The relative abundances of wb1-P19 varied among samples and caves. All the wall biofilms showed relative abundances over 1% and in Ninfa reached up to 13.99%, while sediments ranged from 0.00 to 12.85%. This lineage was absent or presented low abundances (0.00–0.01%) in the water microbial aggregates. The *Acidithiobacillaceae* 9M32 was present in only one sediment sample from Tiberio (27.32%) and absent in the other cave samples. Similarly, *Methylibium* was only observed in Tiberio (0.10% and 6.33%) and *Chujaibacter* in a sediment sample from Ninfa (5.08%) and from Tiberio (5.89%).

The uncultured *Halothiobacillaceae* was only retrieved from the three water microbial aggregates from Befana (ranging between 9.26 and 28.90%). In the same way, *Thiobacillus* was observed with high abundances in the three water microbial aggregates but absent or with low abundances in the rest of samples. In contrast, the gammaproteobacterial KF-JG30-C25 was absent in the water microbial aggregates and only showed significant abundances in sediment from Ninfa (12.33%) and Tiberio (5.09%), in other samples the abundances were below 0.5%.

Of particular importance were the high abundances in water microbial aggregates of the campylobacterial *Sulfurovum* (up to 19.16%), *Sulfurimonas* (up to 13.74%), and *Sulfurospirillum* (up to 6.99%). Other bacteria from the phyla *Desulfobacterota* and *Bacteroidota* were present only in the water microbial aggregates from Befana: *Desulfocapsa* (up to 16.87%), the family *Flavobacteriaceae* (up to 11.96%) and unclassified *Bacteroidetes* BD2-2 (up to 6.19%).

## Discussion

Microbial community analyses showed a strong variety among the samples in terms of richness and alpha diversity indexes without a specific trend in terms of location and nature of samples. On the other hand, beta diversity analysis grouped samples based on their type/nature and collection site, i.e. sediment on the ground, biofilms in the water and biofilms on the walls. This result can be associated with the similar conditions (exposure to nutrients, pH, etc.) that can be present on walls, waters, and ground of different caves carved in the same rock substratum. These conditions could also shape the morphology of the biosignatures; indeed, we observed similar biofilms on walls of the different caves. The mineral substrata along with the specific environmental factors characterizing different niches was demonstrated to significantly influence microbial diversity and structuring microbial community in different zones of the same cave and in diverse caves hosted in the same rock [15, 41, 42].

As regards taxonomy analysis, the almost exclusive high percentage of *Bacteria* (> 99%) over *Archaea* (< 1%) found in the white biofilms and spring water microbial aggregates as well as in the sediments of the three gypsum caves is commonly observed in other epigenic cave studies [43–46]. *Archaea* do not play a role in the white biofilms as denotes its absence, and their abundance in sediments is negligible, despite the abundance reported in surface soils [47]. It is possible that epigenic caves do not possess the required environmental conditions for the successful colonization and thriving of *Archaea*.

### Abundance of Bacteria in Spring Water Microbial Aggregates

The extraordinary number of sulfur bacteria with abundances over 1% (*Sulfurovum*, *Sulfurimonas*, *Sulfurospirillum*) and below 1% (*Thiomicrorhabdus*, *Sulfuricurvum*, *Sulfurifustis*,* Desulfomonile*, *Desulfuromonas*, uncultured *Arcobacteraceae*, *Thiomonas*, *Thiovirga*, *Halothiobacillus*, etc.) is due to the peculiar nature of the cave water with high sulfate content.

The high abundances of *Halothiobacillaceae* (*Gammaproteobacteria*), *Sulfurovum*,* Sulfurospirillum*, *Desulfocapsa*, *Thiobacillus*,* Sulfurimonas* (*Campylobacteria*), *Bacteroidetes* BD2-2, and uncultured *Flavobacteriaceae* (*Bacteroidia*) in the water microbial aggregates from Befana are remarkable. This, together with the occurrence of other sulfur-oxidizing and sulfur-reducing bacteria, agrees with the composition of the floating microbial aggregates analyzed in the thermal hypogenic (sulfuric acid) Fetida Cave [15, 48]. Nevertheless, the genus *Arcobacter*, which comprised almost half of the bacterial population in Fetida Cave, is absent in Befana, and *Sulfurovum* and *Sulfurimonas* with lower abundances in Fetida Cave were the most abundant genera in Befana.

Differences in the composition of microbial mats from the Frasassi Cave system with respect to Befana microbial aggregates were also found. In fact, in Frasassi, the mats are mainly composed of filamentous *Beggiatoa* and *Desulfonema* that were not found in Befana, but other common genera to Frasassi and Befana caves were *Sulfurovum*, *Sulfurimonas*, *Desulfocapsa*, *Thiobacillus*, *Lutibacter*, *Bacteroidetes* BD2-2, *Flavobacterium*, *Desulfobacterium*,* Syntrophus*, and *Sulfuricurvum* [49, 50]. It should be noted that the morphology of all these bacteria found in Befana is rod-shaped and that filamentous bacteria such as *Thiothrix* and uncultured *Arcobacteraceae* presented relative abundances between 0 and 2% in the microbial aggregates. This is consistent with the SEM and confocal data, which show a predominant distribution of rod-shaped and a lower presence of filamentous bacteria.

The microbial aggregates from Befana showed evidence of filamentous sulfur and this is associated to the occurrence of colorless sulfur-oxidizing bacteria (SOB). In the marine environment, hydrogen sulfide is used by SOB as those described in the springs of the gypsum caves. SOB excretes elemental sulfur as spherical globules or sulfur filaments [51]. At mid-ocean ridges following magmatic eruptions, filamentous sulfur appears in the form of flocs that emerge during “snowblower” events [52]. Other authors stated that in the water column, EPS contributes to the formation of flocs (marine snow) [53].

The genera *Sulfurovum* and *Sulfurimonas* are metabolically diverse, microaerophilic chemolithoautotrophs putatively involved in sulfur and nitrate metabolism, as well as hydrogen oxidation [54]. The uncultured *Halothiobacillaceae* belong to a bacterial family characterized by an obligate chemolithoautotrophy using inorganic sulfur compounds or elemental sulfur as electron donors. Members of the *Halothiobacillaceae* occur in seawater, marine sediments, hydrothermal vents, springs, and mine tailings [55, 56]. In Pozzo dei Cristalli stream in Frasassi Cave, a microbial community mainly composed of *Sulfurovum* and *Halothiobacillaceae* was discovered [57].

Although members of bacterial groups involved in sulfur metabolism are typically associated with marine habitats (e.g. *Sulfurovum*, *Sulfurimonas*, *Arcobacter*, *Thiohalophilus*, *Halothiobacillus*), they also occur in different environments such as snowblower vents, springs, mine tailings, and oil reservoirs [55, 58–61], and in white filaments in thermal sulfuric acid caves [15, 48]. Nosalova et al. [62] found a clear dominance of the *Gammaproteobacteria* and *Campylobacteria* classes in terrestrial cold sulfur springs in Slovakia. *Thiothrix* and *Sulfurovum* were identified as the core microbiota of these springs.

Headd and Engel [63] studied the bacterial diversity of white, filamentous microbial mats collected from seven sulfidic springs ranging from 7.5 to 45.3 °C in the USA in order to test if geochemically similar springs, regardless of geographic distance, shared a core microbiome. The authors found that the springs were dominated by *Campylobacteria* and *Gammaproteobacteria*, but there were also high numbers of bacteria that were unique to the springs within these taxonomic groups. The *Bacteroidota* were also prevalent. They concluded that a core sulfidic spring microbiome becomes apparent when comparing the distributions of campylobacterial and gammaproteobacterial groups, but the community composition of each white microbial mat is specific to the spring conditions in which the groups are found and regulated, in part, by the geochemistry at each sulfidic spring. Temperature was identified as an important control on microbial community distribution which could explain the difference found in Befana, Fetida, and Frasassi with respect to the most abundant taxa.

### Abundance of Bacteria in Wall Biofilms

The morphology of the wall biofilms is similar to that reported in other limestone caves [46]. *Crossiella* was the dominant genus in the white biofilms from the walls of Ninfa Cave, as well as in other caves worldwide [64, 65], and particularly in biofilms of different colors [45, 46, 66]. Martin-Pozas et al. [64] stated that in the last 5 years, *Crossiella* was found in different environments, mainly caves, soils, plant rhizospheres, building stones, etc. Surprisingly, the terrestrial biofilms of gypsum caves did not show a high abundance of uncultured *Euzebyaceae*, one of the most abundant genera in white biofilms of Altamira Cave walls [67]. This supports the idea that there are different types of white biofilms in wall caves. This could explain the different composition found between the biofilm of Befana compared to Ninfa.

Previous studies in limestone caves highlight the importance of *Crossiella* in mineral-induced precipitation and the production of antibacterial and antifungal compounds [36, 68]. In calcite moonmilk, *Crossiella* reached a high abundance [36, 42]. This high abundance and evidence from isotopic analysis and culture-mediated precipitation with production of witherite (barium carbonate) and struvite (magnesium ammonium phosphate) indicate that this genus may play an active role in limestone mineral formation [64]. However, to the best of our knowledge, this has not been studied in gypsum environments, but we suspect that *Crossiella* has the same behavior as in limestone caves.

Gonzalez-Pimentel et al. [68] reported the isolation of two strains of *Crossiella* from the walls of Altamira Cave, Spain, showing inhibition of pathogenic Gram-positive and Gram-negative bacteria, and fungi. The presence of gene clusters involved in the synthesis of polyketides, lasso peptides, lanthipeptides, and non-ribosomal peptides point to *Crossiella* representing a source of antibacterial and antifungal compounds. In fact, the presence of high abundances of *Crossiella* in the biofilms from Altamira and Pindal caves, and the absence of fungi in their complex microbial community, support the hypothesis that the biofilm microbiome, mainly composed of *Actinomycetota*, protected the walls from secondary fungal colonization.

The lineage wb1-p19 is the second most abundant bacterium classified as a genus in the wall biofilms from limestone caves, and also in those of Ninfa Cave. A review on the occurrence of the gammaproteobacterial wb1-P19 in caves and other environments delivered a high number of papers describing its presence in different caves and soils. The first report on the lineage wb1-P19 corresponded to a clone retrieved from aquatic microbial mats collected in the Weebubbie Cave, Nullarbor, Australia, by Holmes et al. [69]. These authors included the clone within the *Gammaproteobacteria*, sharing 90.6% of identity with *Thioalkalovibrio denitrificans* af126545, and inferred for this phylotype a chemolithoautotrophic physiology, as it branches within the sulfur or nitrite-oxidizing autotrophic *Gammaproteobacteria*. Later, Schabereiter-Gurtner et al. [70] reported two clones obtained from a white-greyish biofilm on the surface near the rock art painting area of Tito Bustillo Cave, Spain, with similarities of 92.4 and 91.0% with the unidentified wb1-P19 from Holmes et al. [69]. Years later with the use of Illumina MiSeq, the lineage wb1-P19 begins to appear from 2019 onwards with high occurrence in cave biofilms, sediments, and speleothems, and less frequently in soils. Different authors found high relative abundances of wb1-P19 in caves distributed worldwide. Ghezzi et al. [42] reported a relative abundance of wb1-P19 amounting up to 39% in bedrock of Grotta Nera in Italy. Martin-Pozas et al. [35, 45, 46] obtained relative abundances above 20% in yellow biofilms and sediments from Pindal Cave, Spain, and 10% in moonmilk samples. Percentages between 10 and 20% were retrieved from cave vermiculations [43], sediments [71], mineral deposits [72], and a stalactite [44]. Frazier [73] found similar abundances in biofilms from Tennessee caves. Because of previous studies, the functional activity of wb1-P19 was described as sulfur or nitrite-oxidizing autotrophic. However, a recent metagenomic study stated that wb1-P19 is an obligate or facultative methanotroph capable of aerobic and anaerobic growth [74]. Martin-Pozas et al. [75] also linked the microbial communities of moonmilk to methane consumption and CO_2_ uptake in the cave, where *Crossiella* and the lineage wb1-P19 were the key functional microbial groups in the moonmilk formation.

Previous studies in limestone caves indicated that core bacteria in microbial terrestrial biofilms were composed of *Crossiella*, wb1-P19, *Nitrospira*, and a few other lineages [45, 66]. Porca et al. [76] arrived to a similar conclusion studying the yellow biofilms from limestone caves in the Czech Republic, Slovenia, and Spain, and concluded that *Pseudonocardiaceae*, *Gammaproteobacteria*, and *Nitrospirota* constituted the core of the microbial communities. The association of wb1-P19 and *Crossiella* suggested by Martin-Pozas et al. [45] in a limestone cave seems possible in Ninfa Cave, and their high relative abundances suggested that, at least, in one of the white biofilms (NF-9I) they could form the core of the microbial community. Comparatively, the four white biofilms from Befana and Ninfa walls exhibited similar genera composition to other biofilms and biominerals from carbonate caves. The biofilms from Ninfa were mainly composed of *Crossiella* (48.28–66.01%) and distinct wb1-P19 percentages (1.65–13.99%), however, the abundance of *Crossiella* in the biofilms from Befana decreased to 0.00–1.24% and 0.01–4.71% for wb1-P19, while *Pseudomonas* abundance was 0.00–27.69% and *Streptomyces* 0.02–5.62%. These data indicated that although the wall biofilms seem to share some common bacterial genera, the variation in abundances must be governed by local constrains. The formation of different biofilms could be explained by the substrata on which they develop, since in the studied gypsum caves, biofilm formation with different bacterial populations occurs on walls covered by mud, pure gypsum, and sulfuric water.

### Abundance of Bacteria in Sediments

In general, the cave sediments showed differences in relative abundance and diversity of most genera. The distinct microbial communities of two sediments from Ninfa (NF-4D) and Tiberio (TB-3C) are remarkable. Both presented high relative abundances of *Mycobacterium*, KF-JG30-C25, *Chujaibacter*, *Acidothermus*, and unknown *Acetobacteraceae*, particularly in the Tiberio sediment TB-3C with the lower pH. The occurrence of these genera in caves and mines is well documented and likely depends on the cave geochemistry [77–84]. For example, Cheng et al. [85] reported that *Acidothermus* showed a positive correlation with SO_4_^2−^ concentration in Heshang Cave, China. In addition, the *Acidithiobacillaceae* 9M32 was only represented in Tiberio sediment with the low pH. Significant *Acidithiobacillaceae* and *Acetobacteraceae* family populations have been found in Rio Tinto water [86], in water and sediments of Sour Lake in Yellowstone [87], and hot springs in the Taupo Volcanic Zone (New Zealand) [88]. The difference in microbial composition with respect to other sediments within the same cave lies in the different microniches and geochemical compositions that each cave presents.

Other genera with random abundances and occurrences in these gypsum caves were *Streptomyces, Pseudomonas*, *Methylibium*, and the *Chloroflexota* S085. *Streptomyces* and *Pseudomonas* are widespread in show caves (e.g. Altamira, Lascaux, etc.). While *Streptomyces* seems to be a common inhabitant of most caves [89, 90], *Pseudomonas* is particularly abundant in touristic caves and is related to contamination episodes [84, 91–93].

The abundance of *Pseudomonas* in six of the nine sediments is remarkable and in one of the sediments from Tiberio Gothic Hall (TB-8H) it reached up to 88.05%. This sediment was collected in the cave area open to visitors and subjected to contamination. This genus was absent or with abundance < 0.05% in sediments from Pindal Cave, but reached 10% in one of the six sediments collected from Castañar Cave, and the rest ranged between 0.1 and 2.7% [94, 95]. Castañar Cave suffered two episodes of contamination in the last 15 years also associated with the presence of visitors [96, 97].

Another relevant genus by its abundance in two sediments from Ninfa (39.85%) and Tiberio (6.63%) was *Mycobacterium*, absent in Befana, but present in five samples from Ninfa and Tiberio with abundances between 0.00 and 0.33%. *Mycobacterium* was not present in Pindal and Castañar caves, or with abundances < 0.1%. However, a methanotrophic *Mycobacterium* was abundant in biofilms from Sulfur Cave in Romania [82], and nontuberculous *Mycobacterium* were found in other European caves [98, 99]. Other genera relevant for their abundances, generally over 5% in two sediments, NF-4D from Ninfa and TB-3C from Tiberio, were KF-JG30-C25, *Chujaibacter* and unknown *Acetobacteraceae*. These two sediments showed at the same time the high abundances of *Mycobacterium*, but not of *Pseudomonas*.

*Methylibium* is a methanotroph commonly found in contaminated environments [100–102]. Its abundance in Tiberio, in a sediment sample (TB-1A), and its absence in the other caves, might indicate episodes of contamination (the cave is visited frequently and open to public). The *Chloroflexota* S085 has been reported from marine environments [103–105], however, it was present in all the cave samples, except in the microbial aggregates from Befana and in one sediment each from Ninfa and Tiberio.

Comparing the data from the sediments of the three gypsum caves with those of two limestone caves (Pindal and Castañar, both in Spain), the phyla *Pseudomonadota*, *Acidobacteriota*, and *Actinomycetota* accounted for 53.3–80.3% of the total relative abundance in the sediments of Pindal Cave, and for 79.1–86.3% in Castañar Cave [45, 95]. In Tiberio sediments, these three phyla accounted for 80.43–96.32%, in Ninfa for 51.40–91.32%, and in Befana for 59.65–64.33%. While the abundances of the three major phyla were relatively homogeneous in both gypsum and limestone caves, the distribution of genera showed significant differences, largely denoting the influence of local environmental conditions and sediment composition.

## Conclusions

The microbial communities from the three Italian gypsum caves are almost exclusively composed of *Bacteria*. *Archaea* are absent in the biofilms and were absent or negligible in the sediments.

*Actinomycetota* and *Pseudomonadota* were the two most abundant phyla in the wall biofilms and sediment samples. On the other hand, *Pseudomonadota* and *Campylobacterota* were dominant in the Befana microbial aggregates, which clearly evidenced a different microbial community composition in Befana microbial aggregates with respect to white biofilms on the rock walls of Befana and Ninfa.

The Befana microbial aggregates also show different abundances and microbial composition in the dominant genera when compared with those of the Italian Frasassi and Fetida sulfuric acid caves. It is suggested that the community composition is specific and related to the geochemistry of each sulfidic spring.

The most abundant genus in the white biofilms from Ninfa walls was *Crossiella* which corresponded with other wall biofilms from limestone and volcanic caves. However, it was absent in the microbial aggregates from Befana. It is suggested that these differences could be related to variations in the cave substrata where biofilms grow. In the sediments of the three caves *Crossiella* was missing or showed very low abundances.

Another interesting genus was wb1-P19 with variable abundances among samples and caves. This genus was commonly abundant and associated to *Crossiella* in biofilms from limestone caves, but in the studied gypsum caves this association is not so clear, and only in a biofilm from Ninfa it seems possible.

Many other genera show random abundances and occurrences in the sediments from the three gypsum caves. Despite these differences, the sediments present a different bacterial microbial population from both wall and water microbial aggregates. Their occurrence depends on the rock and sediment composition and no uniform pattern was observed, except the presence of acidophilic bacteria in Tiberio sediment with acid pH. The variability in the geochemical analyses of the sediments, environmental conditions, and spatial variability support the wide difference found across the samples.

## Supplementary Information

Below is the link to the electronic supplementary material.

ESM 1 (DOCX 1.93 MB)

## Data Availability

The gene sequences and accompanying metadata were deposited in the Sequence Read Archive (SRA) of NCBI under the project number PRJNA1245447.
