# Supplementary material for: Microbial and Geochemical Variability in Sediments and Biofilms from Italian Gypsum Caves
Source: Microb Ecol. 2025 Jul 24;88:81. doi: 10.1007/s00248-025-02576-3 (PMC12289794; doi:10.1007/s00248-025-02576-3)
Supplement: Supplementary file 1 [file 248_2025_2576_MOESM1_ESM.docx]

**SUPPLEMENTARY INFORMATION**

**MICROBIAL AND GEOCHEMICAL VARIABILITY IN SEDIMENTS AND BIOFILMS FROM ITALIAN GYPSUM CAVES**

**Tamara Martin-Pozas^1^, Daniele Ghezzi^2^, Ilenia M. D’Angeli^3^, Giuliana Madonia^4,5^, Veronica Chiarini^6^, Marco Vattano^7^, Jo De Waele^8^, Martina Cappelletti^2^, Cesareo Saiz-Jimenez^1^, Valme Jurado^1^**

^1^ Instituto de Recursos Naturales y Agrobiologia, IRNAS-CSIC, 41012 Sevilla, Spain

^2^ Dipartimento di Farmacia e Biotecnologie (FaBit), Bologna, 40126 Bologna, Italy

^3^ Istituto Italiano di Speleologia, 40126 Bologna, Italy

^4^ Dipartimento di Scienze della Terra e del Mare, Università degli Studi di Palermo, 90123 Palermo, Italy

^5^ National Biodiversity Future Center (NBFC), 90123 Palermo, Italy.

^6^ Dipartimento di Geoscienze, Università di Padova, 35131 Padova, Italy

^7^ Le Taddarite Naturalistic and Speleological Association, 90141 Palermo, Italy

^8^ Dipartimento di Scienze Biologiche, Geologiche ed Ambientali, Università di Bologna, 40126 Bologna, Italy

Supplementary Figure S1. A. Sediment TB-3C from Re Tiberio Cave. B. Spring water microbial aggregates BF-3C from Befana Cave. C. Wall white biofilm BF-7G from Befana Cave. D. Wall white biofilm NF-3C from Santa Ninfa Cave.

Supplementary Figure S2. Map of Re Tiberio Cave with sampling areas.

Supplementary Figure S3. Map of Befana Cave with sampling areas.

Supplementary Figure S4. Map of Santa Ninfa Cave with sampling areas.

**Supplementary Figure S5.** EDS analysis of water microbial aggregates. A. Calcium sulfate. B. Sulfur filaments. C. Sulfur globules.

**Supplementary Figure S6. Confocal images of water** microbial aggregates**. Right panels show DNA stained with SYTO Deep Red, while left panels show unstained negative controls.**

Supplementary Table S1. Geochemical analysis of sediment and gypsum samples.

| **Sample** | **Type** | **pH** | **EC (mS/cm)** | **CaCO_3_ (%)** | **Organic C (%)** | **Organic Matter (%)** | **N Kjeldahl (%)** | **NO_3_^–^ (mg/kg)** | **NH₄⁺ (mg/kg)** | **Phosphorous (mg/kg)** | **Sulfur**  **(ppm)** | **Iron (ppm)** | **CEC (cmol/kg)** |
| --- | --- | --- | --- | --- | --- | --- | --- | --- | --- | --- | --- | --- | --- |
| NF-1A | Sediment | 7.88 | 2.43 | 19.8 | 0.34 | 0.59 | 0.04 | 4.40 | 35.2 | 5.30 | 88667.55 | 6748.29 | 8.54 |
| NF-3C | Gypsum | 6.59 | 2.43 | 0.80 | 0.08 | 0.14 | 0.004 | 0.60 | 5.7 | 1.20 | 127592.61 | 275.34 | 0.68 |
| NF-4D | Sediment | 6.28 | 2.47 | 0.72 | 0.29 | 0.51 | 0.16 | 12.0 | 52.5 | 16.3 | 120046.55 | 16914.79 | 7.20 |
| NF-5E | Sediment | 8.12 | 2.52 | 53.9 | 0.46 | 0.79 | 0.05 | 133 | 63.5 | 20.0 | 18483.86 | 16634.70 | 12.7 |
| NF-7G | Sediment | 7.55 | 2.39 | 39.4 | 1.02 | 1.76 | 0.11 | 57.2 | 25.4 | 7.80 | 14982.87 | 21768.99 | 23.0 |
| NF-8H | Sediment | 8.08 | 2.33 | 43.8 | 0.40 | 0.69 | 0.04 | 3.1 | 19.4 | 7.20 | 3534.44 | 12321.24 | 11.2 |
| NF-10J | Gypsum | 7.67 | 1.63 | 0.80 | 0.06 | 0.11 | 0.003 | 0.90 | 4.9 | 1.60 | 115426.89 | 357.26 | 0.37 |
| BF-2B | Sediment | 7.78 | 2.81 | 21.4 | 0.40 | 0.68 | 0.04 | 2.60 | 3.6 | 8.70 | 84448.94 | 16736.40 | 14.9 |
| BF-4D | Sediment | 7.75 | 2.30 | 16.5 | 0.37 | 0.64 | 0.05 | 4.00 | 5.0 | 5.60 | 55618.71 | 26255.42 | 17.9 |
| BF-6F | Sediment | 7.74 | 2.18 | 25.0 | 0.34 | 0.58 | 0.05 | 2.20 | 6.6 | 1.90 | 5125.50 | 29509.94 | 18.1 |
| BF-8H | Sediment | 7.73 | 1.47 | 27.5 | 0.27 | 0.46 | 0.05 | 1.30 | 6.8 | 1.90 | 4489.53 | 32038.22 | 19.4 |
| TB-1A | Sediment | 7.75 | 1.18 | 1.09 | 0.03 | 0.06 | 0.03 | 0.80 | 6.6 | 3.60 | 812.63 | 30469.23 | 26.4 |
| TB-2B | Sediment | 6.00 | 2.81 | 2.96 | 0.39 | 0.67 | 0.08 | 447 | 6.6 | 227 | 5377.14 | 34335.62 | 44.4 |
| TB-3C | Sediment | 3.77 | 2.82 | 1.25 | 0.21 | 0.36 | 0.03 | 1030 | 12.2 | 309 | 3346.49 | 33697.97 | 35.7 |
| TB-6F | Sediment | 4.15 | 2.67 | 1.09 | 0.52 | 0.89 | 0.11 | 71.6 | 18.1 | 300 | 16020.66 | 38156.61 | 55.4 |
| TB-8H | Sediment | 5.57 | 2.45 | 1.33 | 0.21 | 0.36 | 0.04 | 4.20 | 10.9 | 72.1 | 17846.14 | 35187.64 | 39.2 |

Supplementary Table S2. Geochemical analysis of water samples.

| **Water samples** | **pH** | **T (C°)** | **CE at 25°C [mS/cm]** | **Na^+^ [ppm]** | **K^+^ [ppm]** | **Ca^2+^ [ppm]** | **Mg^2+^ [ppm]** | **F^-^**  **[ppm]** | **Cl^-^**  **[ppm]** | **NO_3_^-^**  **[ppm]** | **HCO_3_^-^**  **[ppm]** | **HS^-^ [ppm]** | **SO_4_^2-^ [ppm]** | **TDS [ppm]** |
| --- | --- | --- | --- | --- | --- | --- | --- | --- | --- | --- | --- | --- | --- | --- |
| **BF-19** | 7.33 | 13.3 | 7420 | 674.9 | 15.1 | 568.2 | 158.1 | 1.7 | 889.3 | 76.6 | 273.7 | 36.2 | 1924.3 | 3907.1 |
| **BF-05** | 7.04 | 13.0 | 7080 | 529.2 | 16.6 | 589.3 | 82.2 | 1.6 | 716.1 | 0.0 | 223.3 | 7.3 | 1699.6 | 3328.8 |

Supplementary Table S3. Chao1, Shannon, and Simpson’s indexes of all the samples analyzed in this work.

| **Sample** | **Chao1** | **Shannon** | **Simpson’s** |
| --- | --- | --- | --- |
| BF-3C | 222 | 3.26 | 0.9 |
| BF-6F | 394 | 4.27 | 0.95 |
| BF-7G | 317 | 3.27 | 0.87 |
| BF-8H | 436 | 4.57 | 0.96 |
| BF-05 | 28 | 2.94 | 0.94 |
| BF-19 | 113 | 3.98 | 0.97 |
| NF-1A | 492 | 4.83 | 0.97 |
| NF-2B | 248 | 2.36 | 0.63 |
| NF-3C | 302 | 2.29 | 0.56 |
| NF-4D | 95 | 2.62 | 0.81 |
| NF-5E | 544 | 5.39 | 0.99 |
| NF-7G | 547 | 5.06 | 0.97 |
| NF-9I | 316 | 2.83 | 0.75 |
| TB-1A | 335 | 4.05 | 0.94 |
| TB-3C | 140 | 2.68 | 0.86 |
| TB-8H | 122 | 0.84 | 0.22 |

Supplementary Table S4. Distribution of kingdoms in gypsum caves.

| **Sample** | **BF-3C** | **BF-6F** | **BF-7G** | **BF-8H** | **BF-05** | **BF-19** | **NF-1A** | **NF-2B** | **NF-3C** | **NF-4D** | **NF-5E** | **NF-7G** | **NF-9I** | **TB-1A** | **TB-3C** | **TB-8H** |
| --- | --- | --- | --- | --- | --- | --- | --- | --- | --- | --- | --- | --- | --- | --- | --- | --- |
| *Bacteria* | 100.00 | 99.38 | 100.00 | 99.87 | 100.00 | 100.00 | 99.98 | 100.00 | 100.00 | 100.00 | 99.90 | 100.00 | 100.00 | 99.83 | 99.98 | 100.00 |
| *Archaea* | 0.00 | 0.62 | 0.00 | 0.13 | 0.00 | 0.00 | 0.02 | 0.00 | 0.00 | 0.00 | 0.10 | 0.00 | 0.00 | 0.17 | 0.02 | 0.00 |
